# Pyruvate Administration Reduces Recurrent/Moderate Hypoglycemia-Induced Cortical Neuron Death in Diabetic Rats

**DOI:** 10.1371/journal.pone.0081523

**Published:** 2013-11-22

**Authors:** Bo Young Choi, Jin Hee Kim, Hyun Jung Kim, Jin Hyuk Yoo, Hong Ki Song, Min Sohn, Seok Joon Won, Sang Won Suh

**Affiliations:** 1 Department of Physiology, Hallym University, College of Medicine, Chuncheon, Korea; 2 Department of Neurology, Hallym University, College of Medicine, Chuncheon, Korea; 3 Department of Nursing, Inha University, Incheon, Korea; 4 Department of Neurology, University of California San Francisco, San Francisco, California, United States of America; Johns Hopkins University, United States of America

## Abstract

Recurrent/moderate (R/M) hypoglycemia is common in type 1 diabetes patients. Moderate hypoglycemia is not life-threatening, but if experienced recurrently it may present several clinical complications. Activated PARP-1 consumes cytosolic NAD, and because NAD is required for glycolysis, hypoglycemia-induced PARP-1 activation may render cells unable to use glucose even when glucose availability is restored. Pyruvate, however, can be metabolized in the absence of cytosolic NAD. We therefore hypothesized that pyruvate may be able to improve the outcome in diabetic rats subjected to insulin-induced R/M hypoglycemia by terminating hypoglycemia with glucose plus pyruvate, as compared with delivering just glucose alone. In an effort to mimic juvenile type 1 diabetes the experiments were conducted in one-month-old young rats that were rendered diabetic by streptozotocin (STZ, 50mg/kg, i.p.) injection. One week after STZ injection, rats were subjected to moderate hypoglycemia by insulin injection (10U/kg, i.p.) without anesthesia for five consecutive days. Pyruvate (500mg/kg) was given by intraperitoneal injection after each R/M hypoglycemia. Three hours after last R/M hypoglycemia, zinc accumulation was evaluated. Three days after R/M hypoglycemia, neuronal death, oxidative stress, microglial activation and GSH concentrations in the cerebral cortex were analyzed. Sparse neuronal death was observed in the cortex. Zinc accumulation, oxidative injury, microglial activation and GSH loss in the cortex after R/M hypoglycemia were all reduced by pyruvate injection. These findings suggest that when delivered alongside glucose, pyruvate may significantly improve the outcome after R/M hypoglycemia by circumventing a sustained impairment in neuronal glucose utilization resulting from PARP-1 activation.

## Introduction

In an effort to use regular insulin injections to maintain blood glucose levels within a normal range, patients with type 1 diabetes are continuously at risk of encountering episodes of recurrent/moderate hypoglycemia [1,2]. In fact, the risk of hypoglycemia is the major factor limiting strict management of blood glucose. Animal studies have demonstrated that wide-spread neuronal death does not occur in the hippocampus unless blood glucose concentration falls below 1 mM or electroencephalographic (EEG) activity remains isoelectric (silent) for at least 10 minutes. However, it is still possible to induce scattered neuronal death in the cerebral cortex when blood glucose concentrations are sustained just above 1 mM [3-6]. Recurrent episodes of moderate hypoglycemia have been linked to decreased perception of the hypoglycemic state and blunted secretion of counter regulatory hormones, phenomena termed 'hypoglycemia unawareness' and 'hypoglycemia-associated autonomic failure' (HAAF), respectively [7-9]. Even moderate hypoglycemia may produce a significant increase in low-frequency EEG activity [10] and impair cognitive function [11] in diabetic patients. Moderate hypoglycemia, defined as low blood glucose levels (below 2 mM blood glucose for more than 2 hr) without the presence of iso-EEG, induces scattered neuronal death in the cerebral cortex [12], but not in the hippocampus [13,14]. Yamada et al. also found that moderate hypoglycemia did not result in hippocampal neuronal death. However, they did find a deficit in the ability to induce long term potentiation (LTP) at CA1 synapses [13]. For this reason, we hypothesized that repetitive episodes of moderate hypoglycemia may induce synaptic injury in the hippocampus, and consequently the development of cognitive impairment. In support of this hypothesis, we recently demonstrated that repetitive episodes of moderate hypoglycemia leads to synaptic injury in the dendritic area of hippocampus in the absence of detectable neuronal somatic injuries [14]. 

The neuronal death resulting from hypoglycemia is not solely a result of energy failure, but rather results from a sequence of events initiated by hypoglycemia/glucose reperfusion. This sequence of events includes release of synaptic glutamate and activation of neuronal glutamate receptors [15-18], the production of reactive oxygen species [19,20], accumulation of intracellular zinc[21], activation of poly(ADP-ribose) polymerase-1 [5,22], and mitochondrial permeability transition [23-25]. Correction of plasma glucose concentration alone does not interrupt this cell death process [5,22]. During hypoglycemia, conditions that favor the depletion of ATP predominate [26]. Combined glutamate/zinc release and translocation of zinc into postsynaptic neurons induce poly (ADP-ribose) polymerase (PARP) activation after hypoglycemia, which results in energy depletion and neurodegeneration. Adding strong support to the deleterious role of zinc-induced-PARP activation in the etiology of hypoglycemia, hypoglycemia-induced hippocampal neuronal death and spatial learning ability impairment were significantly spared by treatment with PARP inhibitors [5] and by zinc chelators [21]. However, the realization of using either PARP inhibitors or zinc chelators as clinically efficacious neuroprotective agents will require further study, both to gain a more precise understanding of their pharmacological effects and to identify efficient delivery methods, as well as to rule out cytotoxicity. Therefore, it is important to identify other means of intervention beyond PARP inhibitors or zinc chelators, which are both non-toxic and easily deliverable in the clinic.

To date, several hypoglycemia experiments have been performed with normoglycemic (non-diabetic) adult rodents; therefore the exact clinical implications of these studies is not readily apparent since moderate hypoglycemia commonly occurs in juvenile type 1 diabetes patients, rather than adults with no history of diabetes or hyperglycemia. Therefore, the present study was conducted using one-month-old young rats that were rendered diabetic by streptozotocin (STZ) injection, which serves as a model for juvenile type 1 diabetes. 

Metabolic substrates that can serve as an alternative to glucose might be relevant to the restoration of metabolic capacity in affected neurons and the prevention of neuronal death after severe hypoglycemia. Previously, both in vitro and in vivo studies have demonstrated that pyruvate or lactate can protect against neuronal death induced by glucose deprivation [27,28] or from hypoglycemia [22,29]. However, the effect of pyruvate as a putative neuroprotective agent against R/M hypoglycemia-induced cortical neuronal damage has not been tested. If the neuroprotective effect of pyruvate is as remarkable in this setting as it has been shown to be in animal models of brain ischemia [30] and acute/severe hypoglycemia [22], pyruvate may also be an ideal neuroprotective agent in the setting of R/M hypoglycemia. Pyruvate is normally transported across the blood-brain barrier at a rate much slower than glucose, but evidence suggests that significant penetration of the brain by pyruvate can be achieved simply by elevating plasma pyruvate concentrations [30]. Therefore, the aim of this study was to determine whether pyruvate, when administered as an adjuvant to glucose after R/M hypoglycemia, could reduce cortical neuronal death. 

## Materials and Methods

### Ethics Statement

 Animal studies were approved by the Committee on Animal Use for Research and Education at Hallym University (protocol # Hallym 2012-91), in compliance with NIH guidelines. Animal sacrifice was performed under isoflurane anesthesia and all efforts were made to minimize suffering.

### Animal handling

Juvenile male Sprague-Dawley rats were used in this study (90 - 100 g, DBL Co). The animals were housed in a temperature- and humidity-controlled environment (22±2 °C, 55±5% and a 12 hr light: 12 hr dark cycle), supplied with Purina diet (Purina, Gyeonggi, Korea) and water *ad libitum*. 

### Type 1 diabetes rat

For the type 1 diabetes model, rats were injected with streptozotocin (STZ, 50 mg/kg, i.p.). One-month-old male Sprague Dawley rats were given by intraperitoneal injection once per day for two consecutive days. Diabetes was defined as fasting blood glucose levels in excess of 200 mg/dl at seven days after the first injection (Table 1). All rats receiving STZ displayed typical symptoms of diabetes such as polyphagia, polydipsia, and polyuria. Despite the presence of certain caveats, the STZ-induced diabetes mellitus (DM) model offers a very effective tool that can be used in most rodents [31]. 

**Table 1:** Effects of pyruvate injection on blood glucose level before- and after- streptozotocine injection.

	**Before streptozotocin injection**	**After streptozotocin Injection**
**STZ+Vehicle (mg/dl, n=5)**	117 ± 0.61	551 ± 1.24
**STZ+Pyruvate (mg/dl, n=5)**	113 ± 0.54	543 ± 1.36

### Recurrent moderate hypoglycemia

One week after STZ injection, R/M hypoglycemia was induced without anesthesia. After overnight fasting, rats received an insulin injection (10 U/kg, i.p.) to induce moderate hypoglycemia, as previously mentioned with minor modification [32]. Blood glucose was determined using an ACCU-CHEK glucometer (Roche, Indianapolis, IN) with blood drawn from the tail vein at thirty minutes intervals after insulin injection. Previously, we defined ‘moderate hypoglycemia’ as sustained (1 hr) blood glucose concentrations between 1 to 2 mM without induction of coma [14]. Accordingly, blood glucose levels were maintained between 1 and 2 mM for one hour after which hypoglycemia was terminated by increasing blood glucose level with intraperitoneal injection of glucose (25%/1 mL/bolus) (Figure S1). This course was repeated for five consecutive days. To investigate the ability of sodium pyruvate to prevent R/M hypoglycemia-induced neuronal death in the cortex, sodium pyruvate was dissolved in 0.9% saline and injected intraperitoneally once per day (500 mg/kg, Sigma, St Louis, MO) at ten minutes after R/M hypoglycemia. Control rats for these studies received equal volumes of vehicle alone. Pyruvate injection itself induced no blood glucose changes, either in sham-operated or in STZ-treated rats (Table 1 and 2). Since the half-life of pyruvate in plasma is about one hour [22], we hypothesize that pyruvate, when administered alongside glucose after R/M hypoglycemia, may reach sustained levels in brain sufficient to reduce neuronal death. 

**Table 2:** Effects of pyruvate injection on blood glucose level in sham-operated rats.

	**Before saline injection**	**After saline injection**
**Sham+Vehicle (mg/dl, n=6)**	113 ± 0.48	114 ± 0.49
**Sham+Pyruvate (mg/dl, n=6)**	112 ± 0.52	112 ± 0.56

### Brain section preparation

Brains were harvested at three days after R/M hypoglycemia. Animals were anesthetized (urethane, 1.5 g/kg, i.p.) and were perfused intracardially with 0.9 % saline followed by 4 % paraformaldehyde (PFA) in 0.1M phosphate buffer (PB, pH 7.4). The brains were removed immediately and postfixed in the same fixative for one hour. The brain tissues were then cryoprotected by submersion in 30 % sucrose at 4°C for two days. Thereafter, the entire brain was frozen and sectioned with a cryostat microtome at 30 μm thicknesses and stored in cryoprotecting solution.

### Detection of neuronal death

To evaluate the presence and quantity of degenerating neurons, brain sections obtained from R/M hypoglycemia and sham-operated animals were stained with Fluoro-Jade B (FJB) [5,33]. Degenerating neurons were detected by illumination under an epifluorescence microscope with a 450 to 490 nm excitation filter and a 515 nm emission filter. To quantify neuronal death, sections were collected every third cut starting from 4.0 mm posterior to bregma and five coronal sections were analyzed from each animal. An observer masked to the treatment condition counted the number of FJB-positive (+) neurons in the parietal cortex and perirhinal cortex from both hemispheres. The mean numbers of FJB (+) neurons from each region were used for statistical analyses [5,21].

### Detection of oxidative injury

Oxidative injury was estimated by evaluating levels of the lipid peroxidation product, 4HNE (4-hydroxy-2-nonenal). Immunostaining with antibodies against 4HNE (Alpha Diagnostic Intl. Inc., San Antonio, TX) was performed as described previously [34]. Tissues were incubated in a mixture of polyclonal rabbit anti-4HNE antiserum (diluted 1:500) in PBS containing 0.3% Triton X-100 overnight at 4 °C. After washing three times for ten minutes each with PBS, sections were then incubated in a mixture of Alexa Fluor 594-conjugated goat anti-rabbit IgG secondary antibody (Molecular Probes, Invitrogen) at a dilution of 1:250 for two hours at room temperature. The sections were washed three times each for ten minutes with PBS, and mounted on gelatin-coated slides. Five coronal sections were collected from each brain by starting 3.0 mm posterior to Bregma, and were taken at 0.3 mm intervals to span the hippocampus. 4HNE signal intensity was expressed as the mean gray value at the level of layer V pyramidal neurons in the parietal and perirhinal cortex by using Image J.

### Immunofluorescence staining

To identify microglial activation, brain sections were immunostained with a mouse antibody to rat CD11b (Serotec, Raleigh, NC) at a 1:1000 dilution and Alexa Fluor 488-conjugated goat anti-mouse IgG secondary antibody (Molecular Probes, Invitrogen) at a dilution of 1:250. Microglial activation was evaluated by a blind observer. Five sections from each animal were evaluated for scoring. Microglial activation criteria were based on the number of CD11b immunoreactive cells, their morphology and intensity [35] [36]. To identify dendritic damage, the sections were immunostained with a mouse monoclonal antibody specific for microtubule-associated protein 2 (MAP2; Millipore Co, Billerica, MA) at a 1:200 dilution and Alexa Fluor 488-conjugated donkey anti-mouse IgG secondary antibody (Molecular Probes, Invitrogen) at a dilution of 1:250. To identify PARP activation, the sections were immunostained with a rabbit polyclonal antibody against poly(ADP-ribose) (RAR, Trevigen, MD) at a 1:200 dilution and Alexa Fluor 594-conjugated donkey anti-rabbit IgG secondary antibody (Molecular Probes, Invitrogen) at a dilution of 1:250. As described above for measurement of the 4HNE, MAP2 and PAR intensity was also measured at the level of layer V pyramidal neurons in the parietal and perirhinal cortex by Image J and was expressed as the mean gray value.

### Immunohistochemistry

For immunohistochemical staining, monoclonal mouse anti-rat CD11b (diluted 1:1000, Serotec, Raleigh, NC) and monoclonal mouse anti-rat RECA-1 (diluted 1:5000, Serotec, Raleigh, NC), diluted in PBS containing 0.3% Triton X-100, were used as the primary antibodies and incubated overnight at 4 °C. The sections were washed three times for ten minutes each with PBS, incubated in biotinylated anti-mouse IgG (Vector, Burlingame, CA) and ABC complex (Vector, Burlingame, CA), diluted 1:250 in the same solution as the primary antiserum. Between the incubations, the tissues were washed with PBS three times for ten minutes each. The immunoreaction was visualized with 3,3=-diaminobenzidine (DAB, Sigma-Aldrich Co., St. Louis, MO, USA) in 0.1 M Tris buffer and mounted on the gelatin-coated slides. The immunoreactions were observed using an Axioscope microscope (Carl Zeiss, Munchen-Hallbergmoos, Germany). RECA-1 immunoreactivity was quantiﬁed using Image J and expressed as % area [37].

### Detection of reduced GSH

To detect the reduced form of GSH in the brain sections, we probed for GSH-N-ethylmaleimide (NEM) adducts on the free-floating coronal sections [38,39]. Brain sections were incubated with 10 mM NEM for four hours at 4°C, washed, and incubated with mouse anti-GS-NEM (diluted 1:100, Millipore). After washing, the sections were incubated with Alexa Fluor 488-conjugated goat anti-mouse IgG (diluted 1:250, Invitrogen) for two hours. The sections were mounted and photographed with a Zeiss confocal microscope. To quantify GSH intensity, individual neurons from the brain section images were selected as regions of interest (ROIs) and measured using Image J. Briefly, to quantify the GSH intensity, the image was loaded into Image J v. 1.47c and converted into an 8-bit image through the menu option Image→Type→8-bit. Then, regions comprising individual neurons in the parietal and perirhinal cortex images were selected as ROIs. The resulting image was then binarized and restricted to the region of measurement for individual neurons. To measure this area, the menu option Analyze→Measure was selected and then signal from individual neurons was expressed as the mean gray value.

### Detection of vesicular and cytoplasmic zinc

Vesicular and intracellular chelatable zinc was detected by the 4.5 μM N-(6-methoxy-8-quinolyl)-para-toluenesulfonamide (TSQ) method [40]. Three hours after the last episode of hypoglycemia, rats were euthanized and brains were removed without perfusion. For the control experiments, sham-operated or STZ-treated brains were harvested at designated times. The brains were then frozen on powered dry ice and cut into coronal sections. Five evenly spaced sections were collected through the hippocampal region of each brain and air-dried. The dried sections were immersed in a solution of TSQ (Molecular Probes, Eugene, OR) in 140 mM sodium barbital and 140 mM sodium acetate buffer (pH 10.5‑11) for sixty seconds, and then rinsed for sixty seconds in 0.9 % saline. TSQ binding was imaged with a fluorescence microscope (Olympus upright microscope IX70, epi-illuminated with 360 nm UV light) and photographed through a 500 long-pass filter using a CCD cooled digital color camera (Hamamatsu Co., Bridgewater, NJ) with Infinity 3 (Lumenera Co, Ottawa, Canada). From the cortical images, the TSQ stained area was measured using Image J 1.47c (NIH). The mean fluorescence intensity within the parietal or perirhinal cortex area was measured and expressed as arbitrary intensity units after subtraction of background fluorescence. Measurements from the five sections were averaged for each “n”.

### Confocal microscopy

Fluorescence signals were detected using a Zeiss LSM 780 confocal imaging system (Zeiss, New York) with a sequential scanning mode for Alexa 488 and 594. Stacks of images (1024 x 1024 pixels) from consecutive slices of 0.66-0.7 μm in thickness were obtained by averaging eleven scans per slice and were processed with ZEN 2010 (Zeiss, New York).

### Data analysis

Data are expressed as mean ± s.e.m. Statistical significance was assessed by ANOVA and post hoc testing was performed using Scheffe’s test. *P*-values less than 0.05 were considered statistically significant. 

## Results

### R/M hypoglycemia-induced cortical neuronal death is prevented by pyruvate

**Figure 1:**
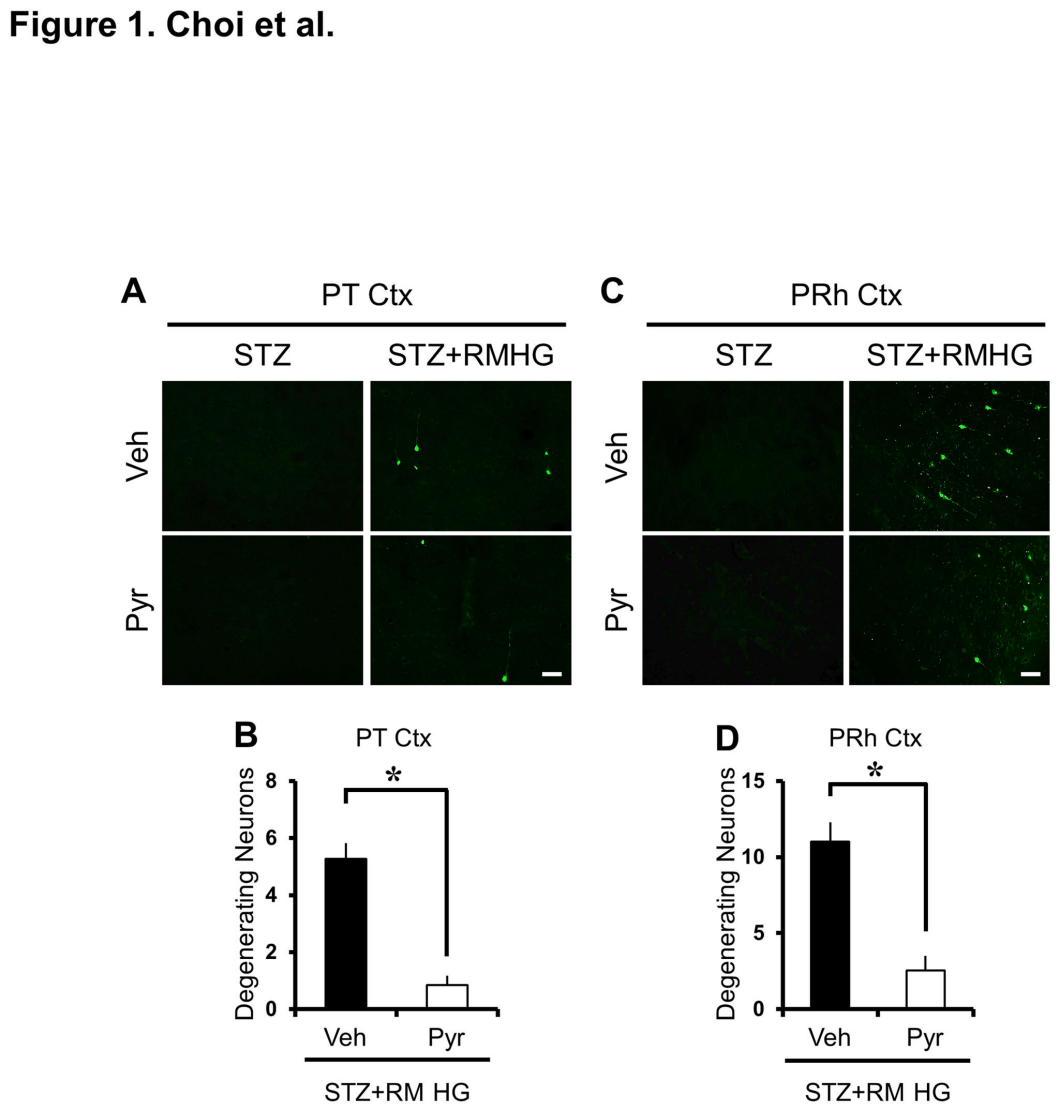
Pyruvate reduces R/M hypoglycemia-induced cortical neuronal death in diabetic rats. (A, C) Fluorescence images show degenerating neurons stained green by Fluoro-Jade B in the parietal (PT Ctx) and perirhinal (PRh Ctx) cortex region at three days after R/M hypoglycemia with or without pyruvate. Pyruvate administered into the intraperitoneal space as an adjuvant to glucose at ten minutes after R/M hypoglycemia and provided protective effects on cortical neuronal death compared to vehicle (glucose only)-treated rats. Scale bar = 100 μm. (B, D) Bar graphs show number of degenerating neurons in five vulnerable brain regions. Data are means ± s.e.m., n=5-6 from each group, *P<0.05.

To test whether pyruvate treatment was neuroprotective following R/M hypoglycemia, rats were sacrificed at three days after insult with or without pyruvate injection. Fluoro-Jade B staining failed to detect degenerating neurons in the control group, with or without pyruvate injection (STZ+Vehicle, n = 5; STZ+Pyruvate, n = 5) but revealed the presence of degenerating neurons in the parietal cortex and perirhinal cortex at three days after R/M hypoglycemia (R/M hypoglycemia+Vehicle, n = 6; R/M hypoglycemia+Pyruvate, n = 6). In rats that received pyruvate in addition to glucose, we observed an 83.9% decrease in FJB (+) neurons in the parietal cortex and a 76.9% decrease in perirhinal cortex (Figure 1).

### R/M hypoglycemia-induced microglial activation in the cortex is reduced by pyruvate

**Figure 2:**
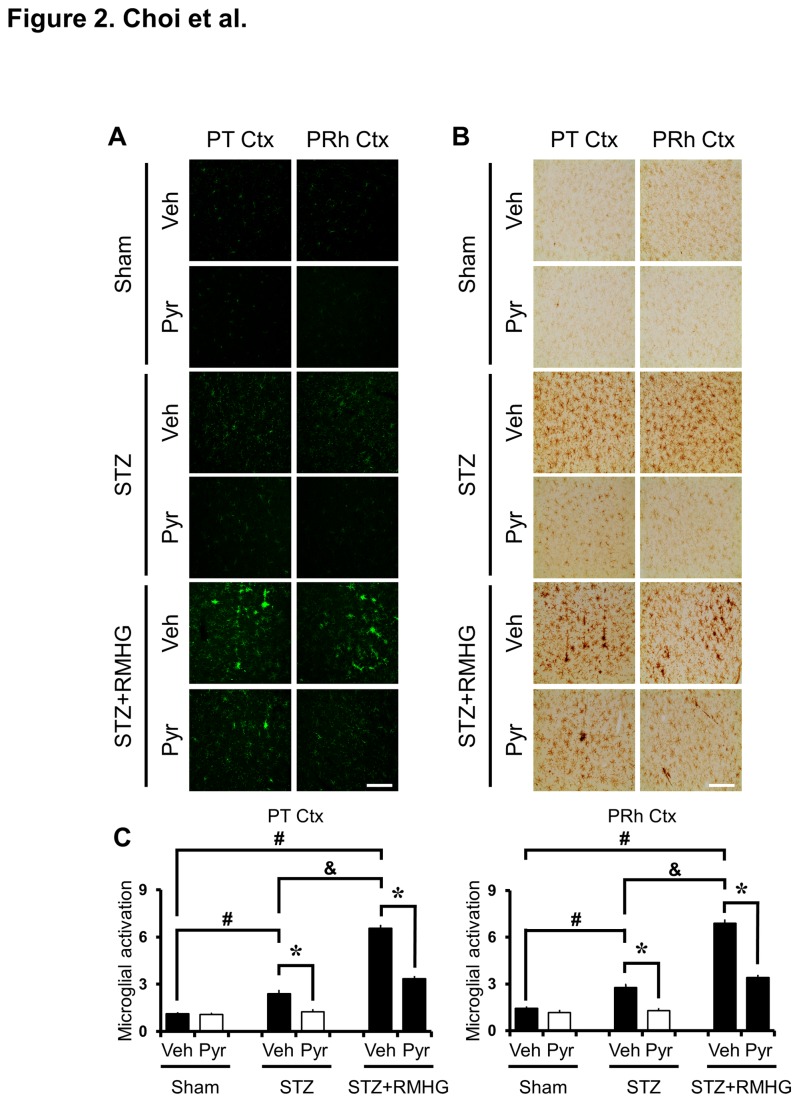
Pyruvate prevents R/M hypoglycemia-induced microglial activation. (A) Fluorescence images show CD11b stained microglia. STZ-treated rats show low intensity CD11b stained microglia in the parietal (PT Ctx) and perirhinal (PRh Ctx) cortex. At three days after R/M hypoglycemia, substantial microglial activation was detected. R/M hypoglycemia-induced microglial number, morphology and intensity are reduced by pyruvate compared to vehicle-treated rats. (B) DAB stained brain sections show that R/M hypoglycemia-induced microglial activation is reduced by pyruvate compared to vehicle-treated rats. Scale bar = 100 μm. (C) Bar graph shows the quantified microglial activation score in the parietal and perirhinal cortex. The quantified score, based on the number of microglia, morphology and intensity, is statistically different between the vehicle-treated rats and the pyruvate-treated rats in the parietal and perirhinal cortex. Data are means ± s.e.m., n=5-6 from each group, *P<0.05.

We tested whether pyruvate administration prevents microglial activation after R/M hypoglycemia by evaluating microglial staining in parietal and perirhinal cortex at three days after R/M hypoglycemia. Brain sections from STZ-treated rats showed ramified morphology and a modest increase in intensity and number of microglia in the cortex compared to sham-operated rats. Pyruvate administration reduced STZ-induced microglial activation. At three days after R/M hypoglycemia, substantial microglial activation was detected in the parietal and perirhinal cortex. Intense microglial activation was prominent in cortical layers Ⅲ-Ⅴ. To test whether pyruvate can exert preventive effects on microglial activation after R/M hypoglycemia, pyruvate was administered into the intraperitoneal space as an adjuvant to glucose at ten minutes after R/M hypoglycemia. The present study found that intraperitoneal administration of pyruvate substantially decreased R/M hypoglycemia-induced microglial activation in the cortex at three days after R/M hypoglycemia in juvenile diabetic rats (Figure 2).

### R/M hypoglycemia-induced cortical oxidative injury is reduced by pyruvate

**Figure 3:**
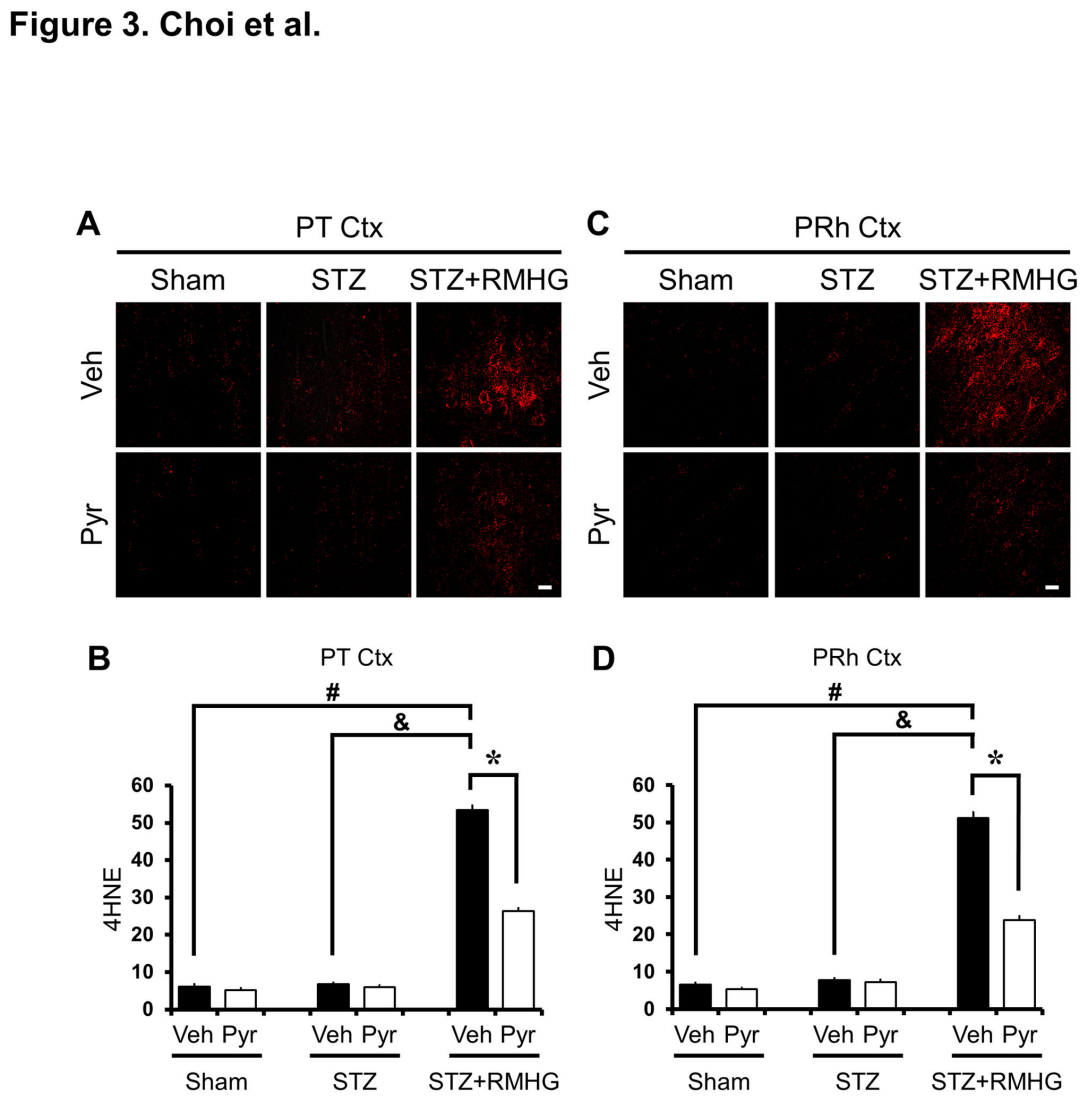
Pyruvate reduces R/M hypoglycemia-induced oxidative injury. (A, C) Fluorescence images show 4HNE (red) antibody staining, indicating oxidative injury in layer V of the the parietal (PT Ctx) and perirhinal (PRh Ctx) cortex. STZ-treated rats showed a slight increase in 4HNE staining in the cortex; 4HNE intensity at three days after R/M hypoglycemia was further increased. Intraperitoneal injection of pyruvate as an adjuvant to glucose at ten minutes after R/M hypoglycemia attenuated 4HNE intensity in the parietal and perirhinal cortex compared to vehicle-treated rats. Scale bar = 20 μm. (B, D) Bar graph represents the 4HNE fluorescence intensity in the parietal and perirhinal cortex as the mean gray value. Data are means ± s.e.m., n=5-6 from each group, *P<0.05.

Rat brain sections were immunohistochemically stained with 4HNE (4-hydroxy-2-nonenal) at three days after R/M hypoglycemia to determine the extent, if any, that oxidative injury had occurred in the cortical neurons. STZ-treated rats showed almost no 4HNE-stained neurons in the cortex as in sham-operated rats. Images taken with a Texas Red filter show several 4HNE (+) neurons in the parietal and perirhinal cortex three days after R/M hypoglycemia. Compared with vehicle-treated rats, pyruvate-treated rats showed a significant reduction in oxidative injury in cortical neurons (Figure 3). Since dendritic injury was also observed in cortical neurons following recurrent moderate hypoglycemia, the increased 4HNE intensity in the cortex may represent oxidative injury to dendrites or glia cells. Our previous study demonstrated that recurrent/moderate hypoglycemia can cause dendritic injury in the hippocampus [14]. Pyruvate injection also decreased the 4HNE fluorescent intensity in both areas.

### R/M hypoglycemia-induced dendrite loss in the cortex is reduced by pyruvate

**Figure 4:**
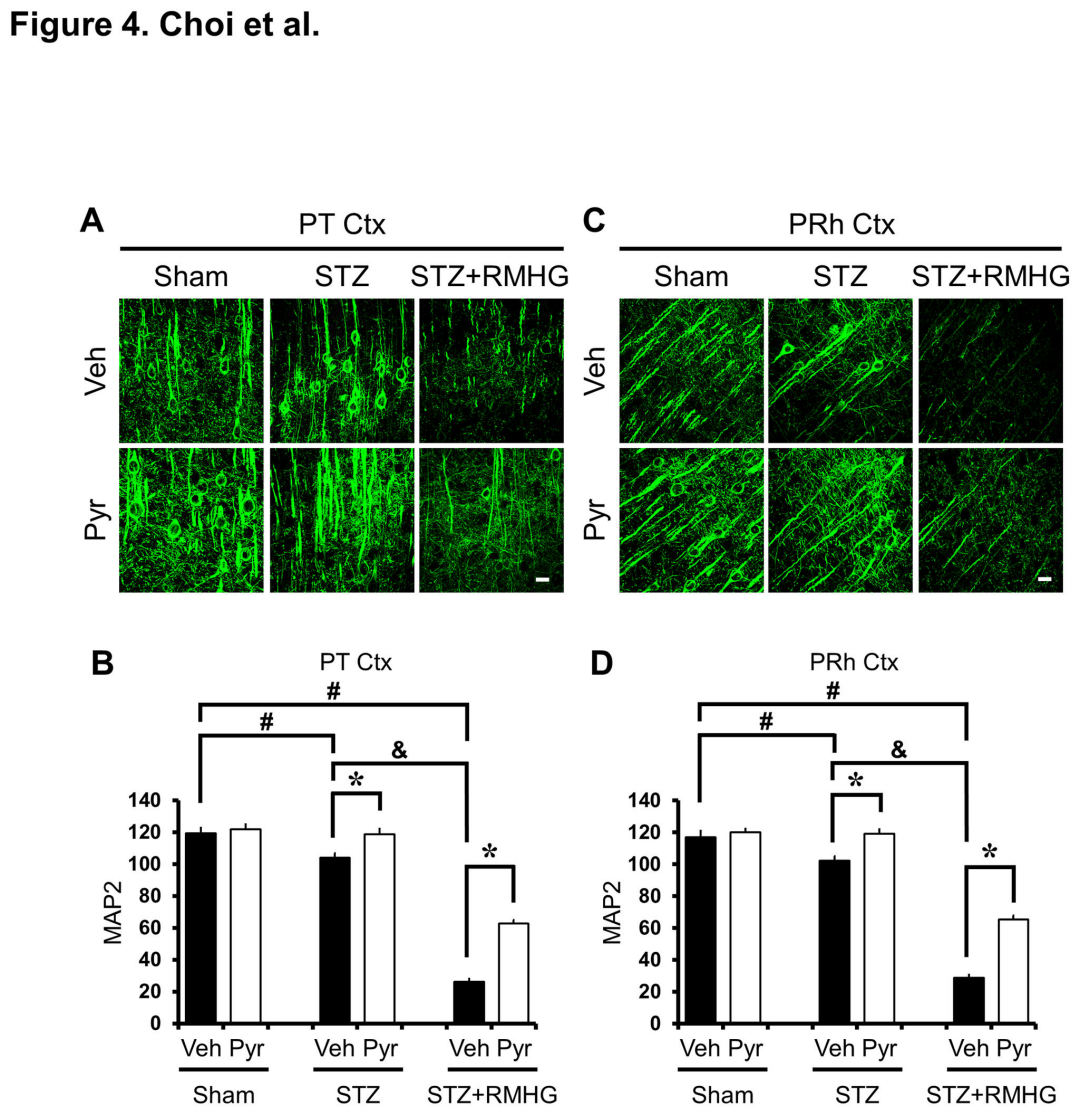
Pyruvate reduces R/M hypoglycemia-induced dendritic damage. (A, C) Fluorescent images show MAP2 (green) antibody stained dendritic structure in layer V of the the parietal (PT Ctx) and perirhinal (PRh Ctx) cortex. The MAP2 intensity in the cortical dendritic area after R/M hypoglycemia was substantially decreased. Intraperitoneal injection of pyruvate as an adjuvant to glucose at ten minutes after R/M hypoglycemia reduced the loss of MAP2 intensity in the parietal and perirhinal cortex compared to vehicle-treated rats. Scale bar = 20 μm. (B, D) Bar graph represents MAP2 fluorescent intensity in the parietal and perirhinal cortex as the mean gray value. Data are means ± s.e.m., n=5-6 from each group, *P<0.05.

To evaluate if R/M hypoglycemia induces changes in cortical dendritic structure, brains were histologically evaluated by MAP2 immunostaining three days after R/M hypoglycemia. Brain sections from STZ-treated rats showed reduced dendritic MAP-2 staining in the cortex compared to sham-operated rats, indicating a loss of dendrites. Pyruvate administration reduced STZ-induced dendrite loss. Interestingly, we found that R/M hypoglycemia reduced MAP2 intensity even further in the parietal cortex and perirhinal cortex. STZ-induced diabetic rats showed significantly reduced MAP2 immunoreactivity in the cortex after RM hypoglycemia compared with pyruvate-treated diabetic rats. R/M hypoglycemia in diabetic rats further decreased MAP2 fluorescent intensity. However, pyruvate administration after R/M hypoglycemia significantly spared this loss of MAP2 intensity (Figure 4).

### Pyruvate inhibits R/M hypoglycemia induced PAR accumulation in the cortex

**Figure 5:**
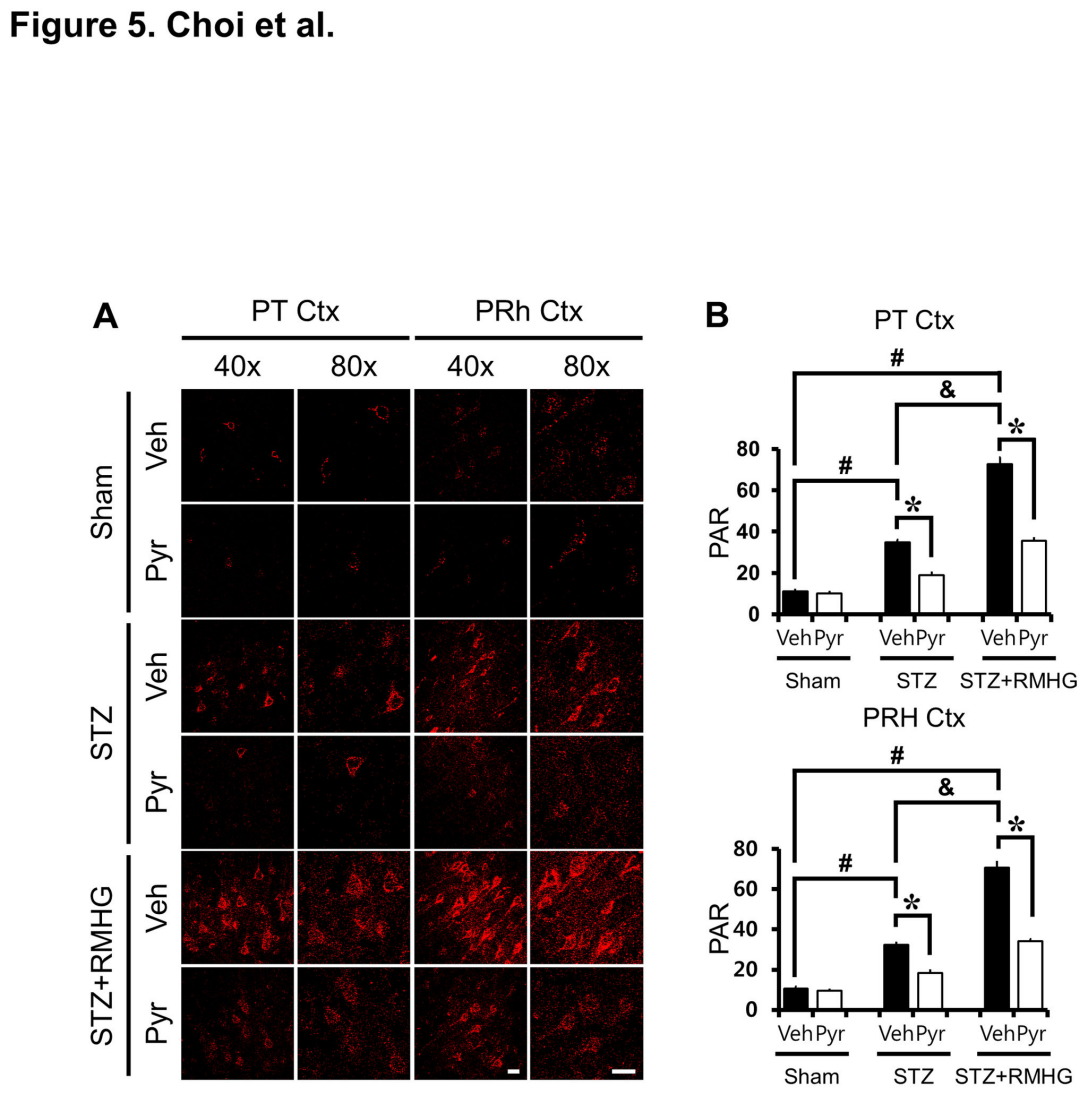
Pyruvate reduces R/M hypoglycemia-induced PAR activation. (A) PARP activation was detected by immunostaining with PAR antibody (red color). Poly (ADP-ribose) (PAR) accumulation in the cortex is shown after STZ treatment. R/M hypoglycemia further increased PAR accumulation in the parietal (PT Ctx) and perirhinal (PRh Ctx) cortex. Compared to vehicle-treated rats, pyruvate-treated rats showed reduced PAR accumulation in cortical neurons. Scale bar = 20 μm. (B) Bar graph represents mean gray value of PAR fluorescent intensity from the parietal and perirhinal cortex. Data are means ± s.e.m., n=5-6 from each group, *P<0.05.

We next examined whether R/M hypoglycemia also induces Poly (ADP-ribose) (PAR) accumulation in the cerebral cortex. Brain sections from STZ-treated rats showed increased PAR accumulation in the cortex compared to sham-operated rats, which was reduced by pyruvate administration. Rats were exposed to R/M hypoglycemia for five days and brains were harvested at three days after the final episode of R/M hypoglycemia. R/M hypoglycemia significantly increased PAR accumulation. Compared with vehicle-treated rats, pyruvate-treated rats showed significantly less cortical PAR accumulation (Figure 5).

### R/M hypoglycemia-induced cortical blood vessel disappearance is reduced by pyruvate

**Figure 6:**
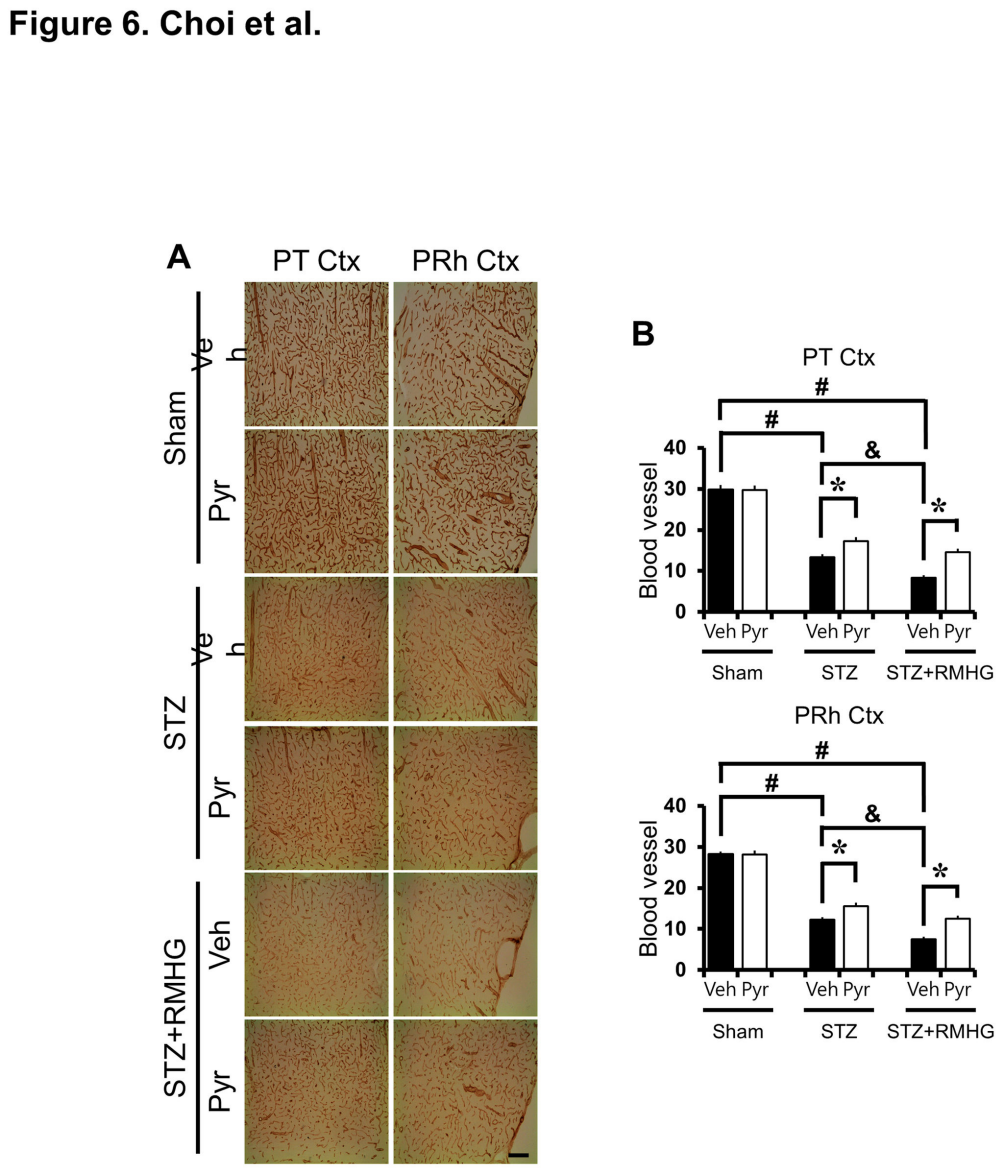
Pyruvate reduces R/M hypoglycemia-induced cortical blood vessel loss. (A) Bright field photomicrographs from coronal sections of cortex demonstrate loss of blood vessels at three days after R/M hypoglycemia by RECA-1 (rat endothelial cell antigen 1) staining. Panels show the progression of blood vessel changes in the parietal (PT Ctx) and perirhinal (PRh Ctx) cortex. After R/M hypoglycemia, blood vessels showed decreased density compared to sham-operated rats. Intraperitoneal injection of pyruvate as an adjuvant to glucose at ten minutes after R/M hypoglycemia reduced blood vessel disappearance. Scale bar = 100 μm. (B) Graph represents the % area of RECA-1 immunoreactivity in the parietal and perirhinal cortex. Data are means ± s.e.m., n=5-6 from each group, *P<0.05.

We found a remarkable decrease in blood vessel diameter by RECA-1 (rat endothelial cell antigen 1) immunostaining in the cortex at three days after R/M hypoglycemia. Particularly in parietal and perirhinal cortex of the cerebral cortex, the blood vessel diameter was reduced and decreased density was observed after R/M hypoglycemia, compared to sham operated animals. The RECA-1 positive blood vessel disappearance was reduced by pyruvate administration after R/M hypoglycemia (Figure 6).

### R/M hypoglycemia-induced neuronal GSH reduction is rescued by pyruvate

**Figure 7:**
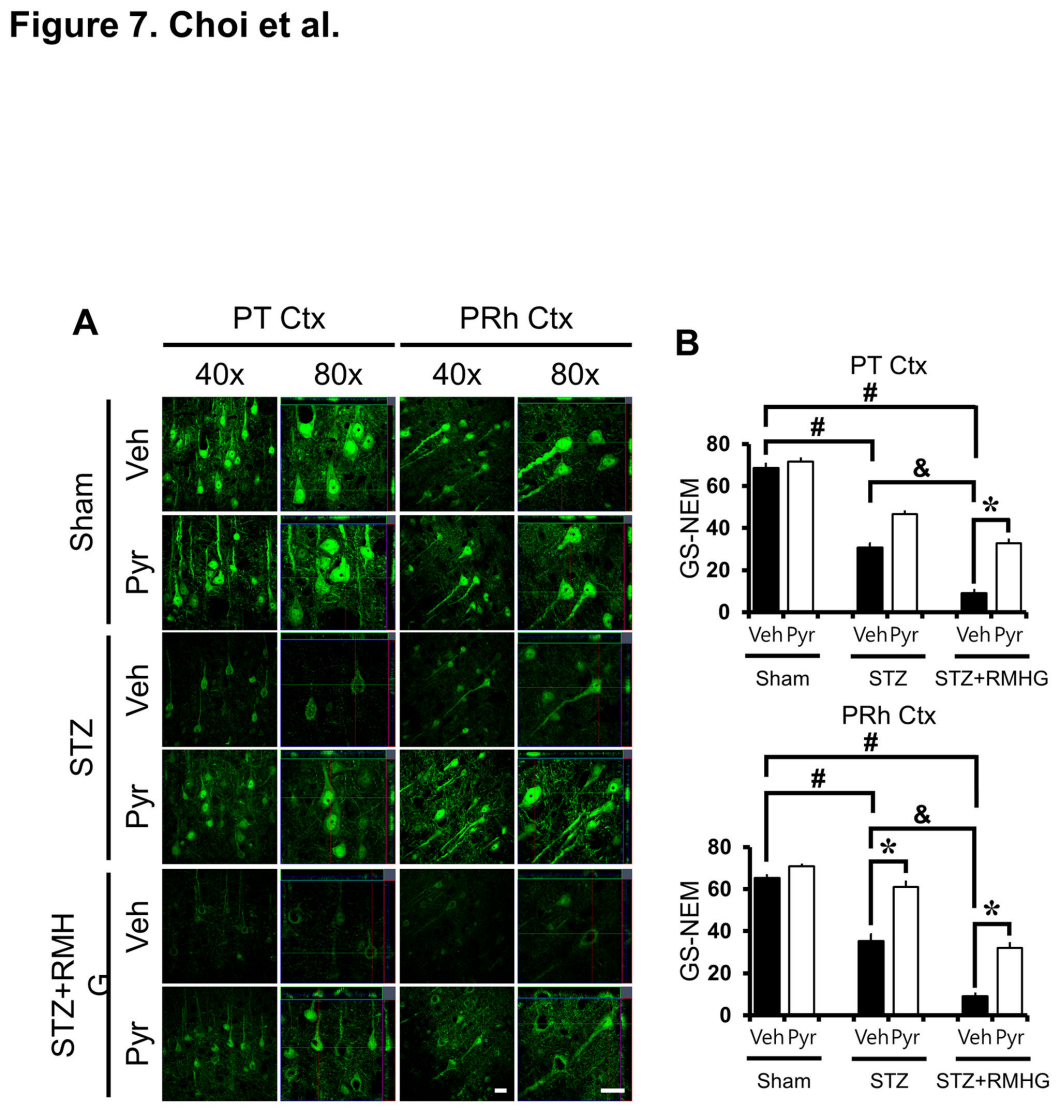
Pyruvate rescues R/M hypoglycemia-induced neuronal GSH loss. (A) R/M hypoglycemia-induced neuronal GSH loss was detected by GS-NEM immunohistochemistry after R/M hypoglycemia, STZ treatment, or sham operation. Representative confocal micrographs show apparent neuronal GSH loss in the parietal (PT Ctx) and perirhinal (PRh Ctx) cortex after R/M hypoglycemia or STZ treatment. R/M hypoglycemia-induced reduction of GS-NEM (+) neuron is reversed by pyruvate injection in the cortex compared to vehicle treatment. Scale bar = 20 μm. (B) Bar graph represents mean gray value of GS-NEM fluorescent intensity from the parietal and perirhinal cortex. Data are means ± s.e.m., n=5-6 from each group, *P<0.05.

In this study, we tested whether R/M hypoglycemia-induced neuronal GSH reduction can be ameliorated by pyruvate administration. To detect the reduced form of GSH, brain sections were histologically evaluated by probing for GSH-N-ethylmaleimide (NEM) adducts at three days after R/M hypoglycemia (Figure 7a). Sham operated animals displayed many GS-NEM (+) neurons in the parietal and perirhinal cortex. However, STZ-induced diabetic rats showed significantly reduced GS-NEM immunoreactivity in the perirhinal cortex compared to pyruvate-treated diabetic rats. R/M hypoglycemia in diabetic rats further decreased GS-NEM fluorescent intensity. However, pyruvate administration after R/M hypoglycemia significantly rescued GS-NEM intensity in the parietal and perirhinal cortex (Figure 7b).

### R/M hypoglycemia-induced zinc accumulation in the cerebral cortex is reduced by pyruvate

**Figure 8:**
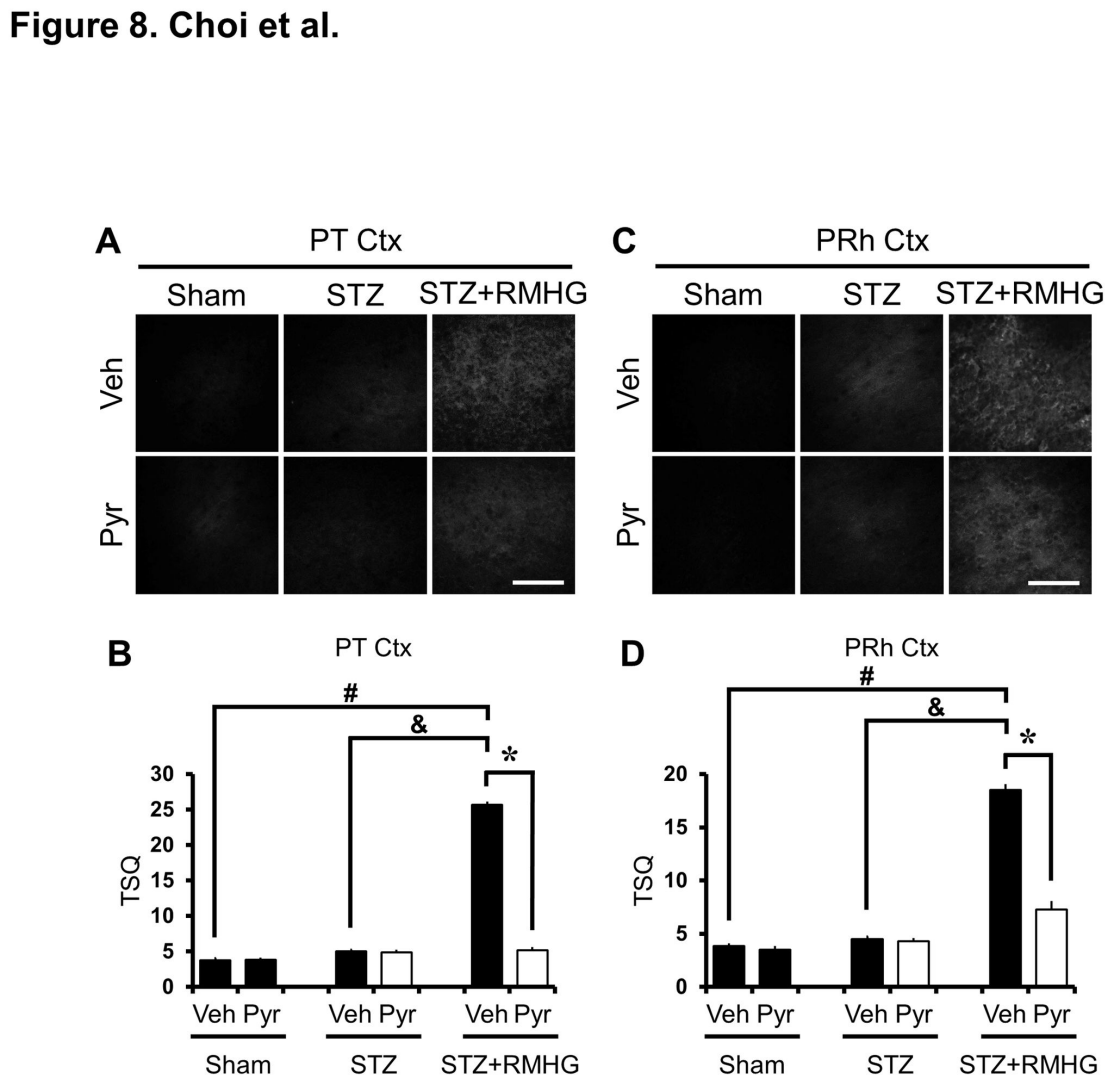
Pyruvate reduces R/M hypoglycemia-induced zinc accumulation. Images show TSQ zinc fluorescence signals in cerebral cortex from vehicle and pyruvate-treated rats after R/M hypoglycemia. R/M hypoglycemia increased the cytoplasmic TSQ signal in the parietal (PT Ctx, A, B) and perirhinal (PRh Ctx, C, D) cortex. Pyruvate-treated rats show reduced TSQ intensity in cortical neurons. Scale bar = 100 μm.

Cytoplasmic zinc in cortical neurons was detected by TSQ staining. In the sham-operated (normoglycemic, no insult) rats, TSQ fluorescence intensity was detected in the outside of cytoplasm of cortical neurons. However, TSQ fluorescence intensity was increased at three hours after last R/M hypoglycemia compared to sham-operated or STZ-treated rats. The intensity of TSQ fluorescence in the cortical neurons was lower in pyruvate-treated rats (Figure 8). 

## Discussion

Here we found the following: first; R/M hypoglycemia induces neuronal death in the cortex of streptozotocin-induced diabetic rats. Second; R/M hypoglycemia induces increased microglial activation, PAR accumulation, BBB disruption and reduces GSH contents in cortical neurons in diabetic rats. Finally; pyruvate administration once per day reduced neuronal death, oxidative injury, microglial activation, BBB disruption and GSH depletion in diabetic rats after R/M hypoglycemia. Therefore R/M hypoglycemia-induced cortical neuronal death in diabetic rats is prevented by pyruvate administration as a supplement alongside glucose.

Mature brain depends on glucose as its primary metabolic substrates. Glucose may be metabolized directly by neurons and glia; alternatively, glucose may be metabolized to lactate in glia and the lactate subsequently shuttled to neurons for oxidative metabolism [41-44]. Lactate, pyruvate and other ketone bodies are similar to glucose in their capacity to support neuronal metabolism in cell culture systems. However, the blood brain barrier in mature brain preferentially transports glucose over other energy substrates. However, pyruvate transport can be enhanced by increasing its plasma concentration. The dose of pyruvate used in the present studies is estimated to achieve a plasma concentration of approximately 5 mM, about 100-fold higher than physiological concentrations. Because pyruvate crosses the blood brain barrier by both facilitated transport and diffusion [45,46], elevating pyruvate to a plasma concentration of 5 mM should lead to enhanced pyruvate transport into brain. Furthermore, using a dosing protocol similar to that employed in the present study, pyruvate has been shown to reduce hippocampal neuronal death after brain ischemia [30] or acute/severe hypoglycemia [22]. 

The neuronal death that results from severe hypoglycemia is not a direct and immediate consequence of low brain glucose availability, but results instead from a cascade of events precipitated by the lack of energy substrate that are themselves not exclusively linked to metabolism. These events include glutamate release, zinc translocation, and PARP-1 activation, all of which have been identified as key steps in the cell death pathway [18,21,47]. Activated PARP-1 consumes cytosolic NAD. The depletion of cytosolic NAD prevents utilization of glucose, and in settings where glucose is the chief metabolic substrate this leads to mitochondrial dysfunction and cell death [48-50]. Cell culture studies have shown that repletion of NAD after PARP-1 activation restores glycolytic capacity and prevents cell death [49,51]. Similarly, providing non-glucose substrates such as pyruvate that can be metabolized without the need for cytosolic NAD also preserves cell viability [48,52]. 

Neuronal zinc accumulation has also been shown to be an important event in this cell death pathway. Zinc has been identified as a trigger for PARP-1 activation [53], and to induce NAD depletion, impaired glycolysis, and cell death in cultured neurons [54]. Hypoglycemia triggers neuronal zinc accumulation *in vivo* and the neurotoxic effects of zinc are blocked by both PARP inhibitors and pyruvate [54]. Furthermore, administration of a zinc chelator in brain can block hypoglycemia-induced PARP-1 activation and neuronal death [21]. Together this supports a model of injury consisting of a sequential series of events by which neuronal zinc release, PARP-1 activation, NAD consumption, and glycolytic blockade contribute to hypoglycemic neuronal cell death. Taken in this context, the present findings suggest that pyruvate promotes neuronal survival after hypoglycemia by bypassing a sustained impairment in glycolysis induced by PARP-1 activation. 

The results of the present studies are similar to those obtained in the acute/severe hypoglycemic brain injury model using pyruvate, in which administration of pyruvate at the termination of hypoglycemia substantially reduced neuronal death in vulnerable brain regions and prevented cognitive impairment [22] and suggest that pyruvate may be an effective substrate for preventing cortical neuronal death and pathological consequences after R/M hypoglycemia, similar to acute/severe hypoglycemia. 

## Supporting Information

Figure S1 **Blood glucose level changes in streptozotocin-treated diabetic rats before-, during- and after-R/M hypoglycemia.** Pyruvate injection showed no effects on blood glucose level. (A) is represented by a table (B) is represented by a graph.
(TIF)
