# Supplementary figures and images for: Pyruvate Administration Reduces Recurrent/Moderate Hypoglycemia-Induced Cortical Neuron Death in Diabetic Rats
Source: PLoS One. 2013 Nov 22;8(11):e81523. doi: 10.1371/journal.pone.0081523 (PMC3838412; doi:10.1371/journal.pone.0081523)

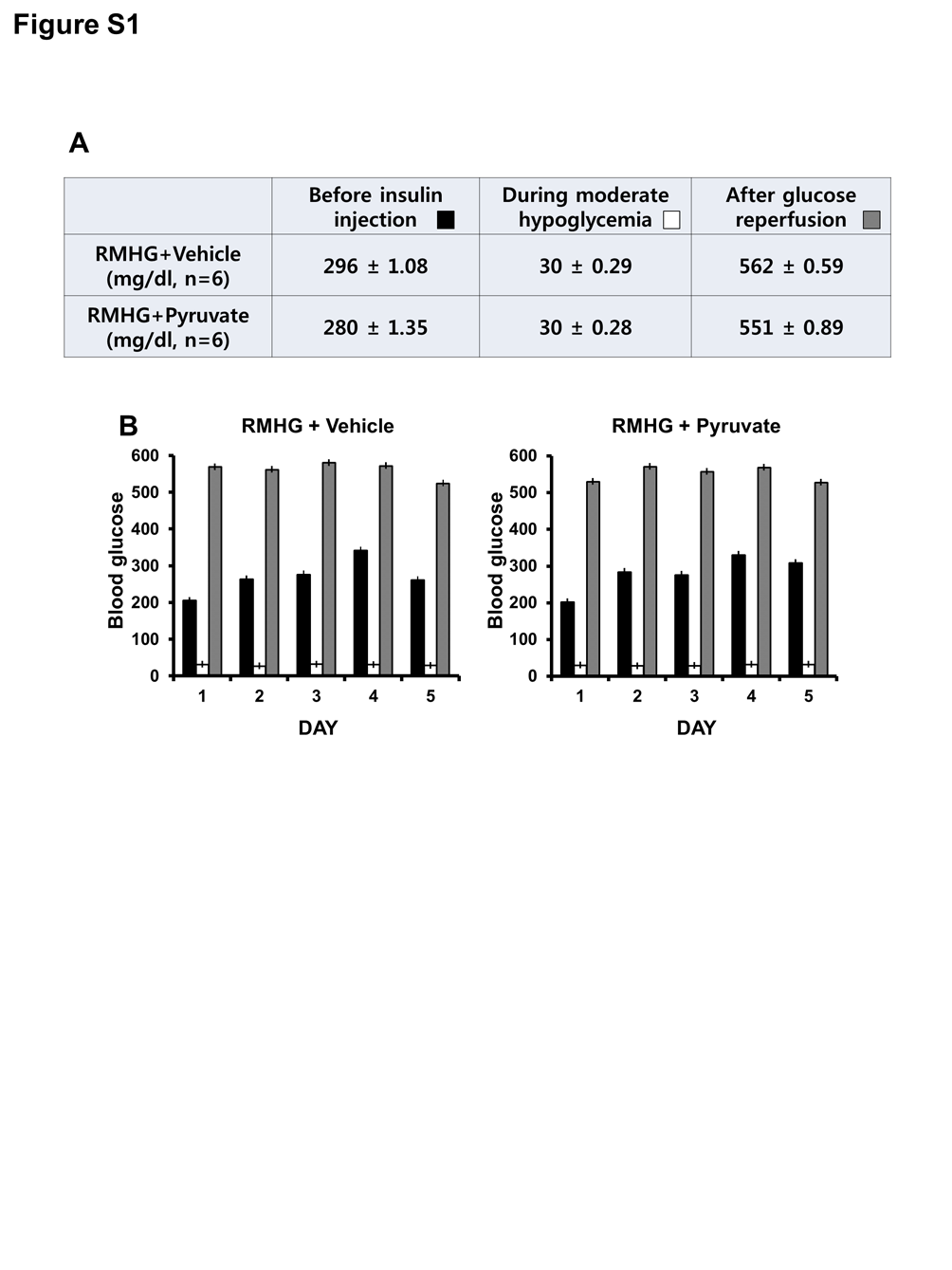

Supplement: Figure S1 — Blood glucose level changes in streptozotocin-treated diabetic rats before-, during- and after-R/M hypoglycemia. Pyruvate injection showed no effects on blood glucose level. (A) is represented by a table (B) is represented by a graph. (TIF) [file pone.0081523.s001.tif]
